# Genomic-Inbreeding Landscape and Selection Signatures in the Polo Argentino Horse Breed

**DOI:** 10.3390/ijms26010026

**Published:** 2024-12-24

**Authors:** Florencia Azcona, Antonio Molina, Sebastián Demyda-Peyrás

**Affiliations:** 1 Cátedra de Medicina Equina, Facultad de Ciencias Veterinarias, Universidad Nacional de La Plata, Calle 60 y 118 s/n, La Plata 1900, Argentina; 2 Consejo Nacional de Investigaciones Científicas y Técnicas (CONICET), CCT La Plata, La Plata 1900, Argentina; 3 Departamento de Genética, Universidad de Córdoba, CN IV KM 396 Edificio Gregor Mendel, 14007 Córdoba, Spain; ge1moala@uco.es

**Keywords:** runs of homozygosity, genomic footprints, ROH island, polo horses, equine genetics

## Abstract

Analyzing genetic variability and inbreeding trends is essential for effective breed management in animal populations. To this, the characterization of runs of homozygosity (ROH) provides a good genomic approach to study the phenomena. The Polo Argentino (PA) breed, globally recognized as the best adapted to playing polo, is known for its strong influence of Thoroughbreds, intense selective breeding, and extensive use of reproductive biotechnologies. This study investigates the PA’s genomic variability, by characterizing the ROH landscape and identifying ROH islands (ROHi) as potential genomic footprints for the breed. PA horses (*n* = 506) were genotyped using EquineGGP™ array v5 (70 k). We calculated the inbreeding coefficient based on ROH (F_ROH_—ancestral and recent) using a chromosomal approach. Finally, we identified genomic regions with increased ROH frequency (ROHi) and their associated genes. An average of 79.5 ROH per horse was detected, with a mean length of 4.6 Mb. The average F_ROH_ was 0.151, but most of them (54%) corresponded to ancestral inbreeding (ROH < 5.5 Mb). However, 4 ROHi were identified in ECA 1, 3, 7 and 17, containing 67 genes, some of which were related to behavior, neurodevelopment, and metabolic functions. This genomic analysis determined, for the first time, the length and location of homozygosity segments in the PA breed and identified ROHi associated with potential genomic regions and genes for positive selection in the breed.

## 1. Introduction

The analysis of the populational structure is a crucial aspect in animal genetics, as it may enhance our understanding of the causes and effects of allelic diversity and distribution. It also allows us to determine the genetic trend followed by a given population over a given period, helping to identify the development of genetic improvement [1]. In addition, population structure analysis facilitates a close control of the inbreeding status, which is a recurring genetic phenomenon in animal production systems [2]. To date, populational structure in animal breeds has been commonly analyzed using pedigree-based approaches, using the data available in the records kept by breeders associations [3]. However, the advance of SNP-based techniques in animal breeding has shifted the methods employed to evaluate populational trends towards genomics [4]. Nowadays, the analysis of genetic variability through genomic data has allowed us to overcome the challenges arising from the use of genealogical data, resulting in a significant improvement in the outcome and practical information provided [5].

In horses, increased inbreeding is particularly important and well-known in some breeds, due to several causes, such as closed enrolment policies, low genetic variability and population sizes, and the extensive use of reproductive assisted biotechnology. While inbreeding is a valuable tool often employed to achieve population uniformity, one of its adverse effects is the increase in the frequency of homozygous recessive alleles [6]. This situation has been associated with a significant decrease in diverse traits related to performance, productivity, fertility, and the increase in the prevalence of genetically related diseases, a phenomenon known as inbreeding depression [7]. This phenotypic drawback tends to be more concerning in species and breeds with small population sizes and/or a closed studbook (where crossbreeding using individuals not registered in the breeding association is not allowed). Both situations are very common in horses.

Over the last few years, the most commonly utilized approach for studying inbreeding in animal populations is the analysis of the distribution and prevalence of runs of homozygosity (ROH) [8]. These are defined as continuous segments of the genome in which an individual is homozygous across all the loci; this allows us to determine a given individual’s molecular-inbreeding value (F_ROH_), as the proportion of the genome covered by ROH. F_ROH_ has been associated with variation in phenotypic traits in several species, including horses [9,10]. In addition, the length of ROH used to estimate inbreeding values is inversely associated with the number of generations elapsed since the inbred event occurred. According to the meiotic recombination theory proposed by Fisher [11], longer ROH derives from a recent common ancestor (recent inbreeding), while shorter ROH stems from a more distant one (ancestral inbreeding). This discrimination is crucial, since ancestral inbreeding often results in reduced inbreeding depression, due to the purging of deleterious alleles occurring across multiple generations [12]. In contrast, recent inbreeding can lead to more deleterious effects, since harmful allele combinations may not be purged effectively within short periods. On the other hand, the presence of regions enriched in runs of homozygosity (ROH), known as ROH islands (ROHi) can signal regions under positive selection [13]. Identifying and characterizing the ROHi-enriched regions is currently one of the most valuable methods for detecting genomic footprints associated with the genetic architecture of specific traits [14,15].

The Polo Argentino (PA) is a breed developed in Argentina for playing polo. Nowadays, PA is the most common breed in high-level polo tournaments worldwide, due to its superior performance. From its origin, PA were heavily influenced by Thoroughbred (TB) horses, a breed with a closed studbook dating back to the 18th century and increased inbreeding levels [16]. In addition, PA is recognized by the widespread use of reproductive biotechnology to obtain high selection intensities in the population, leading to an accelerated reduction in genetic variability, even though the PA breed has an open enrolment policy [17]. Both conditions could lead to increased inbreeding values in the breed, but they may also help to establish selection footprints on specific genomic regions associated with traits of interest for playing polo. To date, no whole-genomic studies have been performed in this breed.

Therefore, this study aimed to investigate, for the first time, the genomic inbreeding landscape in the PA horse breed. To achieve this, we characterized the ROH presence across the whole genome of a large population of PA horses. In addition, we identified the ROHi landscape and performed a functional analysis to determine both candidate genes and the processes associated with selection signatures in Polo horses.

## 2. Results

### 2.1. Heterozygosity and H-W Equilibrium Analysis

Observed (Ho) and expected (He) heterozygosity in the population were calculated. Mean Ho was 0.323, ranging from 0.3 (ECA 1 and ECA 9) to 0.35 (ECA 26). Similar values were determined for He (0.32), ranging from 0.29 (ECA 9) to 0.35 (ECA 26). Mean SNP *p*-values per chromosome were in the Hardy–Weinberg Equilibrium (*p* > 0.05).

### 2.2. ROH Characterization

The ROH landscape characterization in the PA breed (number and length) is shown in Table 1. In total, 40,239 ROH (79.5 per individual) were detected, with an average length of 4.6 Mb, ranging from 1.19 Mb to 68.6 Mb, with most (76%) being shorter than 5.5 Mb. However, nearly 75% of individuals presented at least one ROH ≥ 16.6 Mb, indicating recent inbreeding events and ongoing genetic selection. At the genomic level, the ROH of all the lengths analyzed were detected in most of the chromosomes, except for ECA 12, 25 and 26, which did not exhibit ROH ≥ 16.6 Mb.

### 2.3. Inbreeding Analysis

The average F_ROH_ value was 0.151 in PA horses, with most of the F_ROH_ explained by the occurrence of short ROH (<5.5 Mb) associated with ancestral inbred matings (F_ROHANC_) (Table 2). However, 27% of the F_ROH_ value was explained by mating occurring within the last six generations, suggesting the existence of increased breeding pressure intensity based on phenotypes.

**Figure 1:**
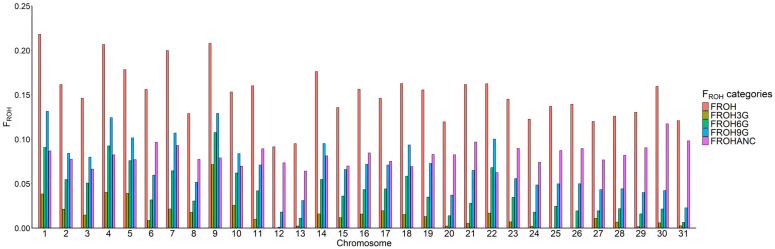
F_ROH_ coefficient by chromosome. Each column represents the F_ROH_ according to the length of the segments considered.

Figure 1 displays the results of the inbreeding analysis categorized by chromosome. F_ROH_ values ranged from 0.09 on ECA12 to 0.21 on ECA1. However, there was considerable variability among individuals. For instance, some horses displayed F_ROH_ levels as high as 80%, while others showed no ROH on certain chromosomes. ECA9 exhibited the highest proportion of ROH segments associated to recent matings (>16.6 Mb, F_ROH3G_ = 0.07), whereas ECA30 had the highest proportion of ancestral ROH. Notably, the F_ROHANC_/F_ROH_ ratio, which reflects the extent of ancestral inbreeding in a genomic region, varied substantially across chromosomes, ranging from 0.38 on ECA9 to 0.81 on ECA31. These results show the existence of non-uniform selection pattern across the genome in the PA breed.

### 2.4. ROHi Identification and Functional Analysis

**Figure 2:**
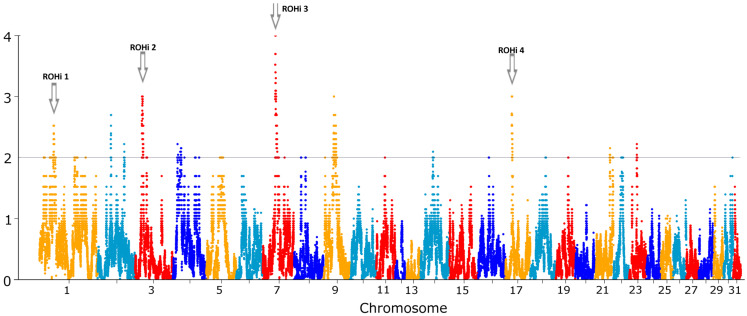
Manhattan plot representing the SNP incidence and the localization of the four ROHi identified in PA breed.

The SNP incidence in ROH averaged 16.3% (with a standard deviation of 8.2%), showing a maximum value of 64.2%. Four significantly ROH-enriched genomic regions were detected (*p* < 0.01, Table 3), spanning 8 Mb and located at ECA1, ECA3, ECA7 and ECA17 (Figure 2).

Gene ontology analysis revealed the presence of 67 genes within the 3 ROHi located on ECA 3, ECA7, and ECA17 (Table 3). In contrast, the ROHi located in ECA1 did not harbor any genes.

According to the literature searches, at least seven of these genes were associated with traits of interest in equines (*OPCML*, *NTM*, *KNCRG*, *JAM3*, *PHF11*, *SETDB2*, and *RCBTB1*). In addition, 17 genes were associated with traits of interest in different species, such as behavior and neuronal function, energy metabolism, musculoskeletal development and growth, reproduction, and the onset of specific diseases. Its functions are discussed below.

## 3. Discussion

In this study, we characterized, for the first time, the genetic variability and inbreeding landscape in a large dataset of Polo Argentino horses using a genomic approach based on the detection of the ROH.

The use of SNP-based genomic approaches in genetic characterizations can effectively overcome the limitations often associated with pedigree data analysis, such as reliability and pedigree depth [5]. Although the PA pedigree is reliable, as the breeders’ association requires a STR-based parentage and individual identification test for each registered animal, the lack of pedigree depth remains a significant factor to consider in population analyses. In addition, in breeds with open studbooks, the continuous addition of individuals from different breeds or of unknown pedigrees is another limitation that can bias the analysis. This is the case for PA horses, where we demonstrated that pedigree analysis leads to a serious underestimation of the actual genomic inbreeding “status” of the individuals [18]. On the contrary, genomic estimations of inbreeding in PA were more similar to those obtained in TB’s, which are the genetic basis of the breed. This result highlights the idea that F_ROH_ is a much more reliable method to estimate an accurate inbreeding level because it can capture identical by descendent segments (IBD) that originated several generations ago. On the other hand, the pedigree-based inbreeding coefficient is a probabilistic method that does not account for the stochastic nature of Mendelian sampling and recombination or changes in allele frequencies due to selection, which can affect its association with the current inbreeding level in a population.

The genomic heterozygosity detected in PA (0.32) was moderate to high in comparison with close-enrollment breeds with reduced populations and/or genetic variability (effective size). For instance, the Pura Raza Español [19], Arabian Horse, and Noriker [20] showed Ho values ranging between 0.23 and 0.28. However, our results were similar to those reported by Petersen, et al. [21] in Thoroughbreds (0.31), even though the PA has an open-enrollment policy and the TB does not. According to Martinez, et al. [22], this could be attributed to the marginal influx of individuals from external breeds or without registration records in PA during the last two decades, contributing to lowering the effective number of parents used. Nevertheless, this is also due to the simple fact that most of the foreign sires and broodmares introduced in the PA are TBs or TB-derived. Since the genetic origin of PA is also closely related to this breed [17], it is expected that the use of TB breeders will have a minor effect on the genomic variability of PA.

ROH-based inbreeding values in the PA breed averaged 15% (F_ROH_ = 0.151). Similar inbreeding levels have been reported in Thoroughbreds [23,24], the primary genetic influence of PA. However, Hill et al. [25] recently reported, in the largest genomic study performed in TBs (*n* = 8950), an average F_ROH_ of 0.28. The lower value observed in PA could be due to the history of the breed itself, which included in its origins an open studbook and native mares as the genetic base. However, it is important to note that the methodology and parameters used can significantly impact the detection of ROH in the genome. In this regard, Meyermans et al. [26] demonstrated that the number of SNP analyzed in the array (medium or high density), the minimum length of the segment, the number of allowed heterozygotes, and the prior data quality control, among others, are parameters that can affect significantly the identification of ROH. Nowadays, we still lack a clear consensus on the optimal parameters to call a ROH for each breed and/or species, despite the efforts being made by the scientific community. Therefore, while trends observed in different studies can be highly informative, comparison of absolute values should be taken with caution. In this context, Schurink et al. [27] analyzed the inbreeding level in nine equine breeds using the same bioinformatic pipeline, finding lowest F_ROH_ values in Warmblood breeds with open pedigrees and gene flow with other populations (0.05–0.097).

F_ROH_ generated by short ROH fragments is associated with inbred matings produced several generations ago, since they were shortened due to the successive meiosis they undergo through the generations [28]. Although inbred matings increase the presence of recessive deleterious alleles in a homozygous state and trigger the onset of inbreeding depression, the natural and artificial selection occurring over the generations elapsed, since the inbred mating can help to purge this negative genetic load. For this reason, it is important to break down the inbreeding coefficient into “ancestral” and “recent” components because it allows us to discriminate between inbreeding values with different ability to produce negative effects on the phenotype [29]. Short ROH that have persisted for several generations in a homozygous state are less likely to contain deleterious alleles associated with inbreeding depression than long ROH segments. If such neutral-effect alleles, in terms of inbreeding depression, are effectively removed through selection, the phenotypic effect should decrease as ancestral inbreeding increases [7]. In this area, Hill et al. [25] found that the probability of TBs debuting in races decreased with an increase in long ROH segments in the genome (>5 Mb), while short ROH (<5 Mb) segments had no phenotypic impact. Similarly, Todd et al. [24] found the ancestral inbreeding coefficient (i.e., the number of times an allele has been identical by descent in an individual’s pedigree) showed a positive association with performance. However, the same authors suggested that while inbreeding and selection had increased the frequency of favorable alleles, they had not effectively eliminated the genetic load in the population. Nearly 55% of the F_ROH_ values detected in PA are produced by short ROH. However, 27% of the F_ROH_ is explained by ROH fragments associated with inbred matings occurring over the last six generations, which is the number of generations elapsed from the origin of PA breed [17]. This value could raise some concerns about the genetic trend of the breed, even more since the widespread use of embryo transfer, and more recently cloned horses, which limits the number of breeding individuals. This increase in recent inbreeding could lead to the onset of an inbreeding depression effect, affecting the phenotype, viability, and fertility on the new generations of PA.

Although the relationship between inbreeding and performance has not yet been assessed in the PA breed, due to the lack of specific phenotypic data, an increase in ancestral inbreeding would not necessarily eliminate the effect of inbreeding depression. Population bottlenecks, which have occurred not only in the origins of the Thoroughbred, are present in the current situation of the PA, helping to maintain undesirable alleles, even in selective sweeps in regions under positive selection [24]. Additionally, the size of the effect of those alleles on performance, together with the intensity of the selection pressure exerted, plays crucial roles in the purging process. This genetic trend is much more effective for alleles that have a significant effect on phenotype, such as lethal alleles, than for those that are only unfavorable for phenotypes [30]. The combination of these minor effects from unfavorable alleles could make the main contribution to the inbreeding depression observed in complex traits, such as those related to sports performance [28]. Furthermore, excessively high rates of inbreeding can favor the fixation of undesirable alleles, outpacing the ability of selection to eliminate them. Therefore, the absence of ROH ≥ 16.6 Mb in some chromosomes may be due to the presence of key genes for development and survival, which do not tolerate the presence of deleterious alleles in a homozygous state [30].

Although ROHs have been distributed across the entire genome, four specific regions located in ECA1, ECA3, ECA7, and ECA17 have been identified as ROH islands (ROHi) due to their high relative frequency in the population. Two of these, ROHi 1 and 2, overlap with genomic regions previously characterized by a low allelic diversity in Thoroughbreds [31]. In contrast, ROHi 4, located on ECA17, overlaps with a region reported as particularly rich in haplotype diversity in Thoroughbreds, which may harbor genes of selective importance in this breed [32]. Finally, ROHi, located in ECA7, has not yet been reported as inbred-enriched in horses. Since ROHi represent common genomic patterns of reduced variability in a given population, pinpointing the potential presence of genes that have undergone positive selection [13], we conducted an analysis of the reported functions of the genes contained within them.

Several of the genes detected within ROHi regions are related to neurological functioning. Across domestication and breed formation, humans have selected optimal behavioral traits to perform specific functions. Similarly, behavioral traits are key selection criteria in horse sport breeds [33]. In Polo horses, temperamental and behavioral traits are particularly crucial, as they must be cooperative and docile, yet alert and responsive to cope with the highly demanding nature of the game [34]. Achieving this combination in a single individual is challenging, and many horses are excluded from playing high-performance tournaments during the training period due to insufficient mental and cognitive ability. Therefore, the selection process performed in PA may have influenced loci linked to the development of nervous system [35]. In this context, preliminary results have pinpointed phenotypic differences in several behavioral traits in PA related to high-level polo performance [36]. However, to our knowledge, no association studies have yet been performed on the topic. Given the well-established genetic basis for the control and development of cognitive traits in horses [37,38], our results may support the idea that these types of traits are among the most important/selected among the PA. Nonetheless, further research is needed in this area.

The *OPCML* and *NTM* genes located within ROHi 3 have been associated with “durability” traits in racehorses [39]. Both genes showed an association with the number of starts at 2 and 3 years, a period of economic significance in the racing career of Thoroughbreds. Both genes are associated with neurobiology, behavior, and nervous system development, and have been linked to intelligence and cognition in humans [40]. In horses, the region harboring these genes was found to be highly differentiated between heavy and light horse breeds, potentially reflecting a selection signature [41]. The authors suggest that the functionality of these genes could potentially explain the large temperamental differences existent between the two biotypes. Similarly, the *KNCRG* gene, located within ROHi 4, encodes a protein involved in regulating voltage-dependent potassium channels, which are related to neuronal excitability and learning. This gene has been also associated with improved racing performance in Norwegian–Swedish Coldblooded Trotter horses [42]. In addition, several of the genes located within the ROHi have been associated with neurodevelopment, learning, behavioral traits or neurologic conditions across different species such as cattle, mice, and humans, including *ATXN1L* [43], *ST3GAL2* [44], *VAC14* [45], MTSS2 [46], *FA2H* [47], *ZNRF1* [48], *IGSF9B* [49,50], *B3GAT1* [51] and *KPNA3* [52]. Even though no association of these genes with cognitive traits in horses has yet been reported, it would be expected that, in some cases, orthologous genes produce a similar effect on the phenotype, thus affecting cognitive traits. Overall, these results support the idea that genetic variants could influence behavior traits (temperament, learning ability, etc.), which directly affect athletic performance, and could have a significant impact on polo horses.

Some of the genes located within ROHi have been previously linked to metabolic and mitochondrial functions. The *NCAPD3* gene encodes the D3 subunit of the Condensin II complex, which plays a significant role in mitochondrial metabolism and oxidative stress [53]. This gene is overexpressed in bulls with high fertility, suggesting a role in sperm functionality [54]. Similarly, the *SPATA19* gene has also been associated with mitochondrial function in the sperm line in mice [55], playing an important role in male fertility [56]. Similarly, *CHST4* and *TAT* were linked to protein metabolism [57,58], while *PHLPP2* and *PDPR* are involved in carbohydrate or lipid metabolic regulation [59,60]. Identifying genes or QTLs associated with traits like growth and reproduction is crucial in horse breeding, as they directly impact the breeder’s economy and efficiency. Additionally, genes involved in energy metabolism and mitochondrial function are al-so putative candidates for controlling muscle activity, energy production, thermoreg-ulation, oxygen utilization, and oxidative stress response. Indeed, mitochondrial res-piratory capacity is a key factor in the control of skeletal muscle physiology and per-formance [61]. All of these factors are crucial in the animal’s adaptation and performance during exercise in horses [62].

Finally, certain genes identified within the ROHi exhibit functions associated with immune responses (*ARL11* [63], *IL34* and *CYSLTR2*) or specific diseases. For instance, a recent study describes the association between *CYSLTR2* gene polymorphisms and asthma in humans [64]. In turn, the *JAM3* gene encodes an adhesion molecule expressed in various tissues, including bronchial smooth muscle. Ben Hamouda et al. [65] found that this gene is under-expressed in the smooth muscle tissue of the bronchi in asthmatic horses, suggesting it may play a significant role in the development of this disease and the accompanying remodeling changes. It has also been proven that good aerobic capacity seems to be a key factor in determining riding performance in horse competitions, allowing the animals to perform and utilize energy sources in a better way [66]. In fact, horses possess a series of structural and functional variations in the respiratory system which improve the oxygen consumption at the muscle and their mitochondria, giving these animals a 2.5-fold higher level of aerobic performance capacity [67]. For this reason, an adequate respiratory function is being used as predictor of future racing performance in TB horses [68]. It is therefore to be expected that PA horses have some genetic advantage to cope with the high metabolic requirements of the game. Since no phenotypic data are available on these types of traits, our results may suggest that one of the adaptations of PA to be considered as the most suitable breed for playing polo could be associated with the respiratory and metabolic function.

## 4. Materials and Methods

### 4.1. Samples

A total of 506 PA horses (129 males and 377 females) belonging to 86 different polo horse breeders from Argentina were analyzed in this study. Hair samples were collected in stud farms, embryo transfer centers, or training centers located in 6 different provinces for subsequent DNA extraction. Sample selection was performed, aiming to include the most significant genetic lines of the PA, according to our previous genetic analysis performed in the breed [17], including direct descendants of several of the key ancestral lines which contribute most to the breed.

### 4.2. DNA Extraction and Genotyping

DNA extraction was taken from hair follicles using the High-Q™ Tissue Genomic DNA Purification Kit (Tiaris Biosciences, Córdoba, Spain), following the manufacturer’s protocol. DNA quality was assessed through standard parameters such as concentration (ng/dL) and purity (A260/280 and A260/230 absorbances), using a Nanodrop™ spectrophotometer (Thermo Fisher Scientific, Madrid, Spain).

Samples were genotyped using the Equine GGP array v5 (Neogen©, Ayr, Scotland). This microarray comprises 71,590 SNPs evenly distributed across the whole genome developed (Neogen, 2023).

#### Genotype Quality Control

The raw data (final report) were first pruned using SNP, and individual call rates using the PLINK V1.9 software [69]. Only SNPs genotyped in at least 90% of the samples and samples with more than 95% of SNPs genotyped were kept. In the final step, only the SNPs located in the 31 autosomes were included in the analysis. As a result, all the 506 PA horses with 64,203 genotyped SNPs were retained in the final dataset.

### 4.3. Genomic Analysis

#### 4.3.1. Estimation of Heterozygosity and H-W Equilibrium in the Population

The analysis included calculating observed (Ho) and expected (He) heterozygosity in the population. The comparison between Ho and He allows us to verify whether a population is in the Hardy–Weinberg (H-W) equilibrium. These parameters were estimated using PLINK.

#### 4.3.2. Inbreeding Coefficient Based on ROH

ROH analysis was performed at a whole-genome level following the best practices described by Meyermans et al. [26]. The analysis was implemented using the sliding window method in the R package DetectRUNS V0.9.6 [70]. ROH calls were determined on autosomal chromosomes, using the following parameters: scanning window size = 52; minimum number of SNP in a run = 52; threshold of overlapping windows to call a SNP in a run = 0.05; maximal gap = 1,000,000 bp; minimum length of a run = 1,000,000 bp; maximum number of heterozygous SNP in the sliding window = 0; maximum number of missing SNP in sliding window = 1; minimal SNP density per segment = 1 SNP per 10 kbps. The minimum number of SNP (L = 52) in both the scanning window and in a ROH segment were determined by the formulae proposed by Lencz et al. [71] and adapted by Purfield et al. [72]:

$$L=\frac{{log}_{e}\frac{\propto }{n_{s}\times n_{i}}}{{log}_{e}(1-het)}$$

where α is the percentage of false positive ROH (set in 0.05), n_s_ is the number of SNPs per individual, n_i_ is the number of individuals genotyped and het the mean heterozygosity in the population.

Subsequently, the inbreeding coefficients per individual (F_ROH_), at both the genomic and chromosomal levels, were determined as follows:

$$F_{ROH}=\frac{\Sigma LROH}{Lgenome}$$

where “ΣLROH” is the sum of the total length of all ROH, and “Lgenome” is the total length of the genome covered by SNPs (2,276,871,601 bp) in total F_ROH_ or the length of each specific chromosome (when calculating F_ROH_ per chromosome). This calculation was performed using the “summary.runs” function of DetectRUNS.

To assess the influence of ancestral and recent inbreeding, the number of generations since the occurrence of consanguineous mating on the F_ROH_ of individuals was determined based on runs of homozygosity (ROH) of specific lengths, following the theory proposed by Fisher (1954). According to this author, the average length (in centimorgans, cM) of homozygous fragments inherited from a common ancestor follows a distribution of 100/2g, where g represents the number of generations since the consanguineous event. We selected 3, 6, and 9 generations arbitrarily for inbreeding analysis. Therefore, assuming 1 cM equals 1 Mb [25], ROH ≥ 16.6 Mb long correspond to inbreeding events within the last three generations and were used for calculating recent inbreeding (called F_ROH3G_), while ROH ≥ 8.3 Mb was generated within the last six generations (F_ROH6G_), and ROH ≥ 5.5 Mb within the last nine generations (F_ROH9G_). Ancestral inbreeding (F_ROHANC_) was estimated as F_ROH_ − F_ROH9G_, reflecting consanguineous events that occurred more than nine generations before.

#### 4.3.3. ROH Island (ROHi) Identification

ROH-enriched genomic regions (ROHi) were identified across the entire population. First, we calculated the frequency of each SNP’s presence within a ROH. Then, we identified the most common ROH segments, defined as those present in at least 50% of the individuals. To assess the significance of SNP incidences, we additionally estimated a *p*-value for each SNP using the statistical methodology described by Goszczynski et al. [73], under the null hypothesis that ROHs are uniformly distributed across the genome. Briefly, we quantified the number of times each SNP occurred within a ROH in the population (SNP incidence) and determined the maximum SNP incidence per chromosome. To generate the test incidence values, a number between 0 and the maximum SNP incidence was randomly generated 1,000,000 times. Thereafter, the *p*-value for each SNP was estimated as the proportion of instances where the actual SNP incidence exceeded the randomized incidence. Finally, ROHi were defined as genomic regions present in ≥50% of individuals and containing at least three SNPs with a *p*-value < 0.01, indicating a 100-fold increase in the likelihood of ROH occurrence in that region compared to random expectation.

#### 4.3.4. Functional Analysis

Functional analysis of ROHi was conducted by intersecting each region with the equine genome assembly (EquCab 3.0) to identify annotated genes located within these regions using the Bioconductor environment [74] and HelloRanges V1.28.0 package [75] in R. The functions of these genes were evaluated by performing a comprehensive literature review using major online databases such as PubMed and Scopus, using keywords like “gene name” and “horse”. In cases where no relevant results were found in equines, gene functions were also determined using orthologous genes in cattle or mice. Finally, candidate genes were selected for discussion if their functions were related to traits influencing athletic performance, behavior, energy metabolism, musculoskeletal conformation, or overall health.

## 5. Conclusions

This study provides the first large-scale genomic characterization of the Polo Argentino (PA) horse breed. We detected moderate-to-high levels of inbreeding in the breed that were underreported in previous pedigree-based analyses, emphasizing the importance of genomic approaches for examining variability in breeds with a limited pedigree depth like PA. Despite the high rate of ancestral inbreeding, nearly a quarter of the total has happened in just the last 6 generations. This recent inbreeding increases the risk of inbreeding depression, which could adversely affect the future of the breed. Finally, we identified four ROH-enriched regions (ROHi) harboring genes linked to key physiological functions relevant to athletic performance in horses. However, further validation through phenotypic data and additional analyses is required prior to integrating our results into breeding programs.

## Figures and Tables

**Table 1:** Number and mean length of runs of homozygosity (ROH) in the Argentine Polo horse population.

ROH Length	Number of ROH	Percentage of Total ROH	Mean Length ± SD (Mb)	Percentage of Horses with ROH
<5.5 Mb	30,760	76%	3 ± 1	100%
≥5.5–<8.3 Mb	5057	13%	6.7 ± 0.8	99.8%
≥8.3–<16.6 Mb	3672	9%	11.1 ± 2.2	100%
≥16.6 Mb	750	2%	22.4 ± 7	75.7%
Total	40,239	100%	4.6 ± 3.8	100%

SD = standard deviation. Mb = megabase.

**Table 2:** Mean of overall and ROH-based inbreeding coefficients of different length (F_ROH_).

	Mean ± SD	Percentage of Total F_ROH_
F_ROH_	0.151 ± 0.03	-
F_ROH3G_	0.011 ± 0.01	7%
F_ROH6G_	0.041 ± 0.02	27%
F_ROH9G_	0.069 ± 0.02	46%
F_ROHANC_	0.082 ± 0.01	54%

F_ROH_ = ROH-based inbreeding; F_ROH3G_ = F_ROH_ produced within the last 3 generations; F_ROH6G_ = F_ROH_ produced within the last 6 generations; F_ROH9G_ = F_ROH_ produced within the last 9 generations; F_ROHANC_ = F_ROH_ produced more than 9 generations ago. SD = standard deviation.

**Table 3:** Genomic regions selected as potential ROHi.

ROHi	ECA	No. SNPs	Start	End	Length (pb)	Mean *p*	Genes
1	1	3	47,620,527	47,855,868	235,341	0.007	No genes found
2	3	91	21,763,318	24,717,282	2,953,964	0.0046	*A0A3Q2H454_HORSE*, *A0A3Q2HI84_HORSE*, *AARS1*, *ATXN1L*, *CALB2*, *CHST4*, *CLEC18B*, *CMTR2*, *COG4*, *DHODH*, *DHX38*, *EXOSC6*, *FA2H*, *FCSK*, *GLG1*, *HYDIN*, *IL34*, *IST1*, *IST1 homolog* (*A0A3Q2HHC8_HORSE*), *IST1 homolog* (*F7DPV9_HORSE*), *LOC100054938*, *MARVELD3*, *MLKL*, *MTSS2*, *PDPR*, *PHLPP2*, *PKD1L3*, *PMFBP1*, *RFWD3*, *SF3B3*, *ST3GAL2*, *TAT*, *TLE7*, *TXNL4B*, *VAC14*, *WDR59*, *ZFHX3*, *ZNF19*, *ZNF23*, *ZNF821*, *ZNRF1*
3	7	64	40,715,678	43,730,750	3,015,072	0.0017	*ACAD8*, *B3GAT1*, *GLB1L2*, *IGSF9B*, *JAM3*, *LOC100072895*, *NCAPD3*, *NTM*, *OPCML*, *RNA-directed DNA polymerase*, *SPATA19*, *THYN1*
4	17	39	20,602,482	22,313,205	1,710,723	0.0044	*ARL11*, *CAB39L*, *CDADC1*, *CYSLTR2*, *EBPL*, *FNDC3A*, *KCNRG*, *KPNA3*, *MLNR*, *PHF11*, *RCBTB1*, *SETDB2*, *SPRYD7*, *TRIM13*

pb = base pair.

## Data Availability

Genomic data analyzed in this study were generated under a collaboration agreement between the AACCP and the research team. The genomic dataset remains confidential since it involves individuals belonging to commercial herds. It can be obtained for scientific purposes upon reasonable request and under a confidentiality agreement by contacting corresponding author.

## References

[1] Machmoum M., Boujenane I., Azelhak R., Badaoui B., Petit D., Piro M. (2020). Genetic Diversity and Population Structure of Arabian Horse Populations Using Microsatellite Markers. J. Equine Vet. Sci..

[2] Cole J.B. (2024). Perspective: Can we actually do anything about inbreeding?. J. Dairy Sci..

[3] Wellmann R., Hartwig S., Bennewitz J. (2012). Optimum contribution selection for conserved populations with historic migration. Genet. Sel. Evol..

[4] Rothschild M.F. (2017). Genomics and genetics: A daily double for the horse industry. Equine Vet. J..

[5] Kardos M., Luikart G., Allendorf F.W. (2015). Measuring individual inbreeding in the age of genomics: Marker-based measures are better than pedigrees. Heredity.

[6] Leroy G. (2014). Inbreeding depression in livestock species: Review and meta-analysis. Anim. Genet..

[7] Charlesworth D., Willis J.H. (2009). The genetics of inbreeding depression. Nat. Rev. Genet..

[8] McQuillan R., Leutenegger A.L., Abdel-Rahman R., Franklin C.S., Pericic M., Barac-Lauc L., Smolej-Narancic N., Janicijevic B., Polasek O., Tenesa A. (2008). Runs of Homozygosity in European Populations. Am. J. Hum. Genet..

[9] Hill E.W., McGivney B.A., MacHugh D.E. (2023). Inbreeding depression and durability in the North American Thoroughbred horse. Anim. Genet..

[10] McGivney B.A., Han H., Corduff L.R., Katz L.M., Tozaki T., MacHugh D.E., Hill E.W. (2020). Genomic inbreeding trends, influential sire lines and selection in the global Thoroughbred horse population. Sci. Rep..

[11] Fisher R.A. (1954). A fuller theory of junctions in inbreeding. Heredity.

[12] Doekes H.P., Veerkamp R.F., Bijma P., de Jong G., Hiemstra S.J., Windig J.J. (2019). Inbreeding depression due to recent and ancient inbreeding in Dutch Holstein-Friesian dairy cattle. Genet. Sel. Evol..

[13] Saravanan K.A., Panigrahi M., Kumar H., Bhushan B., Dutt T., Mishra B.P. (2020). Selection signatures in livestock genome: A review of concepts, approaches and applications. Livest. Sci..

[14] Grilz-Seger G., Druml T., Neuditschko M., Mesarič M., Cotman M., Brem G. (2019). Analysis of ROH patterns in the Noriker horse breed reveals signatures of selection for coat color and body size. Anim. Genet..

[15] Laseca N., Molina A., Ramón M., Valera M., Azcona F., Encina A., Demyda-Peyrás S. (2022). Fine-Scale Analysis of Runs of Homozygosity Islands Affecting Fertility in Mares. Front. Vet. Sci..

[16] Bower M.A., McGivney B.A., Campana M.G., Gu J., Andersson L.S., Barrett E., Davis C.R., Mikko S., Stock F., Voronkova V. (2012). The genetic origin and history of speed in the Thoroughbred racehorse. Nat. Commun..

[17] Azcona F., Valera M., Molina A., Trigo P., Peral-Garcia P., Sole M., Demyda-Peyras S. (2020). Impact of reproductive biotechnologies on genetic variability of Argentine Polo horses. Livest. Sci..

[18] Azcona F., Molina Alcalá A., Peral Garcia P., Demyda-Peyrás S. Genomic data reveals a serious underestimation of pedigree inbreeding levels in Polo Argentino horses. Proceedings of the 2021 International Society for Animal Genetics Meeting.

[19] Poyato-Bonilla J., Laseca N., Demyda Peyrás S., Molina Alcalá A., Valera M. (2021). 500 years of breeding in the Carthusian Strain of Pura Raza Español horse: An evolutional analysis using genealogical and genomic data. J. Anim. Breed. Genet..

[20] Druml T., Neuditschko M., Grilz-Seger G., Horna M., Ricard A., Mesaric M., Cotman M., Pausch H., Brem G. (2018). Population Networks Associated with Runs of Homozygosity Reveal New Insights into the Breeding History of the Haflinger Horse. J. Hered..

[21] Petersen J.L., Mickelson J.R., Cothran E.G., Andersson L.S., Axelsson J., Bailey E., Bannasch D., Binns M.M., Borges A.S., Brama P. (2013). Genetic Diversity in the Modern Horse Illustrated from Genome-Wide SNP Data. PLoS ONE.

[22] Martinez M.M., Costa M., Corva P.M. (2021). Analysis of Genetic Variability in the Argentine Polo Horse With a Panel of Microsatellite Markers. J. Equine Vet. Sci..

[23] Cunningham E.P., Dooley J.J., Splan R.K., Bradley D.G. (2001). Microsatellite diversity, pedigree relatedness and the contributions of founder lineages to thoroughbred horses. Anim. Genet..

[24] Todd E., Ho S., Thomson P., Ang R., Velie B., Hamilton N. (2018). Founder-specific inbreeding depression affects racing performance in Thoroughbred horses. Sci. Rep..

[25] Hill E.W., Stoffel M.A., McGivney B.A., MacHugh D.E., Pemberton J.M. (2022). Inbreeding depression and the probability of racing in the Thoroughbred horse. Proc. Biol. Sci..

[26] Meyermans R., Gorssen W., Buys N., Janssens S. (2020). How to study runs of homozygosity using PLINK? A guide for analyzing medium density SNP data in livestock and pet species. BMC Genom..

[27] Schurink A., Shrestha M., Eriksson S., Bosse M., Bovenhuis H., Back W., Johansson A.M., Ducro B.J. (2019). The Genomic Makeup of Nine Horse Populations Sampled in the Netherlands. Genes.

[28] Sumreddee P., Hay E.H., Toghiani S., Roberts A., Aggrey S.E., Rekaya R. (2021). Grid search approach to discriminate between old and recent inbreeding using phenotypic, pedigree and genomic information. BMC Genom..

[29] Baumung R., Farkas J., Boichard D., Mészáros G., Sölkner J., Curik I. (2015). grain: A computer program to calculate ancestral and partial inbreeding coefficients using a gene dropping approach. J. Anim. Breed. Genet..

[30] Hedrick P.W., Garcia-Dorado A. (2016). Understanding Inbreeding Depression, Purging, and Genetic Rescue. Trends Ecol. Evol..

[31] Fawcett J.A., Sato F., Sakamoto T., Iwasaki W.M., Tozaki T., Innan H. (2019). Genome-wide SNP analysis of Japanese Thoroughbred racehorses. PLoS ONE.

[32] Petersen J.L., Mickelson J.R., Rendahl A.K., Valberg S.J., Andersson L.S., Axelsson J., Bailey E., Bannasch D., Binns M.M., Borges A.S. (2013). Genome-Wide Analysis Reveals Selection for Important Traits in Domestic Horse Breeds. PLoS Genet..

[33] Yokomori T., Ohnuma A., Tozaki T., Segawa T., Itou T. (2023). Identification of Personality-Related Candidate Genes in Thoroughbred Racehorses Using a Bioinformatics-Based Approach Involving Functionally Annotated Human Genes. Animals.

[34] Azcona F., Karlau A., Trigo P., Molina A., Demyda-Peyrás S. (2024). Genomic tools for early selection among Thoroughbreds and Polo Argentino horses for practicing polo. J. Equine Vet. Sci..

[35] Wickens C., Brooks S.A. (2020). Genetics of Equine Behavioral Traits. Vet. Clin. N. Am. Equine Pract..

[36] Álvarez R.P., Demyda Peyrás S., Prado Silva R.H., Arroyo P., Trigo P.I. Análisis de componentes principales en las etapas clasificatorias de una prueba de doma. Proceedings of the XXXIII Conferencias Internacionales de Veterinaria Equina, FCV UNR.

[37] Galizzi Vecchiotti G., Galanti R. (1986). Evidence of heredity of cribbing, weaving and stall-walking in thoroughbred horses. Livest. Prod. Sci..

[38] Yokomori T., Tozaki T., Ohnuma A., Ishimaru M., Sato F., Hori Y., Segawa T., Itou T. (2024). Non-Synonymous Substitutions in Cadherin 13, Solute Carrier Family 6 Member 4, and Monoamine Oxidase A Genes are Associated with Personality Traits in Thoroughbred Horses. Behav. Genet..

[39] McGivney B.A., Hernandez B., Katz L.M., MacHugh D.E., McGovern S.P., Parnell A.C., Wiencko H.L., Hill E.W. (2019). A genomic prediction model for racecourse starts in the Thoroughbred horse. Anim. Genet..

[40] Pan Y., Wang K.S., Aragam N. (2011). NTM and NR3C2 polymorphisms influencing intelligence: Family-based association studies. Prog. Neuro-Psychopharmacol. Biol. Psychiatry.

[41] Gurgul A., Jasielczuk I., Semik-Gurgul E., Pawlina-Tyszko K., Stefaniuk-Szmukier M., Szmatola T., Polak G., Tomczyk-Wrona I., Bugno-Poniewierska M. (2019). A genome-wide scan for diversifying selection signatures in selected horse breeds. PLoS ONE.

[42] Velie B.D., Fegraeus K.J., Solé M., Rosengren M.K., Røed K.H., Ihler C.-F., Strand E., Lindgren G. (2018). A genome-wide association study for harness racing success in the Norwegian-Swedish coldblooded trotter reveals genes for learning and energy metabolism. BMC Genet..

[43] Felício D., du Mérac T.R., Amorim A., Martins S. (2023). Functional implications of paralog genes in polyglutamine spinocerebellar ataxias. Hum. Genet..

[44] Sturgill E.R., Aoki K., Lopez P.H., Colacurcio D., Vajn K., Lorenzini I., Majić S., Yang W.H., Heffer M., Tiemeyer M. (2012). Biosynthesis of the major brain gangliosides GD1a and GT1b. Glycobiology.

[45] Cao X., Lenk G.M., Mikusevic V., Mindell J.A., Meisler M.H. (2023). The chloride antiporter CLCN7 is a modifier of lysosome dysfunction in FIG4 and VAC14 mutants. PLoS Genet..

[46] Corona-Rivera J.R., Zenteno J.C., Ordoñez-Labastida V., Cruz-Cruz J.P., Cortés-Pastrana R.C., Peña-Padilla C., Bobadilla-Morales L., Corona-Rivera A., Martínez-Herrera A. (2023). MTSS2-related neurodevelopmental disorder: Further delineation of the phenotype. Eur. J. Med. Genet..

[47] Araujo A.C., Carneiro P.L.S., Alvarenga A.B., Oliveira H.R., Miller S.P., Retallick K., Brito L.F. (2021). Haplotype-Based Single-Step GWAS for Yearling Temperament in American Angus Cattle. Genes.

[48] Wakatsuki S., Araki T. (2023). Novel insights into the mechanism of reactive oxygen species-mediated neurodegeneration. Neural Regen. Res..

[49] Babaev O., Cruces-Solis H., Piletti Chatain C., Hammer M., Wenger S., Ali H., Karalis N., de Hoz L., Schlüter O.M., Yanagawa Y. (2018). IgSF9b regulates anxiety behaviors through effects on centromedial amygdala inhibitory synapses. Nat. Commun..

[50] Coleman J.R.I., Bryois J., Gaspar H.A., Jansen P.R., Savage J.E., Skene N., Plomin R., Muñoz-Manchado A.B., Linnarsson S., Crawford G. (2019). Biological annotation of genetic loci associated with intelligence in a meta-analysis of 87,740 individuals. Mol. Psychiatry.

[51] Zeng L., Ming C., Li Y., Su L.Y., Su Y.H., Otecko N.O., Liu H.Q., Wang M.S., Yao Y.G., Li H.P. (2017). Rapid Evolution of Genes Involved in Learning and Energy Metabolism for Domestication of the Laboratory Rat. Mol. Biol. Evol..

[52] Aomine Y., Sakurai K., Macpherson T., Ozawa T., Miyamoto Y., Yoneda Y., Oka M., Hikida T. (2022). Importin α3 (KPNA3) Deficiency Augments Effortful Reward-Seeking Behavior in Mice. Front. Neurosci..

[53] Deutschman E., Ward J.R., Kumar A., Ray G., Welch N., Lemieux M.E., Dasarathy S., Longworth M.S. (2019). Condensin II protein dysfunction impacts mitochondrial respiration and mitochondrial oxidative stress responses. J. Cell Sci..

[54] Muhammad Aslam M.K., Sharma V.K., Pandey S., Kumaresan A., Srinivasan A., Datta T.K., Mohanty T.K., Yadav S. (2018). Identification of biomarker candidates for fertility in spermatozoa of crossbred bulls through comparative proteomics. Theriogenology.

[55] Mi Y., Shi Z., Li J. (2015). Spata19 is critical for sperm mitochondrial function and male fertility. Mol. Reprod. Dev..

[56] Obholz K.L., Akopyan A., Waymire K.G., MacGregor G.R. (2006). FNDC3A is required for adhesion between spermatids and Sertoli cells. Dev. Biol..

[57] Bi Y., He Y., Huang J.Y., Xu L., Tang N., He T.C., Feng T. (2013). Induced maturation of hepatic progenitor cells in vitro. Braz. J. Med. Biol. Res..

[58] Müller D., Kuiper H., Böneker C., Mömke S., Drögemüller C., Chowdhary B.P., Distl O. (2005). Assignment of BGLAP, BMP2, CHST4, SLC1A3, SLC4A1, SLC9A5 and SLC20A1 to equine chromosomes by FISH and confirmation by RH mapping. Anim. Genet..

[59] Kim K., Kang J.K., Jung Y.H., Lee S.B., Rametta R., Dongiovanni P., Valenti L., Pajvani U.B. (2021). Adipocyte PHLPP2 inhibition prevents obesity-induced fatty liver. Nat. Commun..

[60] Yan J., Lawson J.E., Reed L.J. (1996). Role of the regulatory subunit of bovine pyruvate dehydrogenase phosphatase. Proc. Natl. Acad. Sci. USA.

[61] Votion D.M., Gnaiger E., Lemieux H., Mouithys-Mickalad A., Serteyn D. (2012). Physical fitness and mitochondrial respiratory capacity in horse skeletal muscle. PLoS ONE.

[62] Littiere T.O., Castro G.H.F., Rodriguez M.d.P.R., Bonafé C.M., Magalhães A.F.B., Faleiros R.R., Vieira J.I.G., Santos C.G., Verardo L.L. (2020). Identification and Functional Annotation of Genes Related to Horses’ Performance: From GWAS to Post-GWAS. Animals.

[63] Arya S.B., Kumar G., Kaur H., Kaur A., Tuli A. (2018). ARL11 regulates lipopolysaccharide-stimulated macrophage activation by promoting mitogen-activated protein kinase (MAPK) signaling. J. Biol. Chem..

[64] Dos S.J.T., Dos S.C.R., Alcântara-Neves N.M., Barreto M.L., Figueiredo C.A. (2019). Variants in the CYSLTR2 are associated with asthma, atopy markers and helminths infections in the Brazilian population. Prostaglandins Leukot. Essent. Fatty Acids.

[65] Ben Hamouda S., Miglino M.A., de Sá Schiavo Matias G., Beauchamp G., Lavoie J.P. (2021). Asthmatic Bronchial Matrices Determine the Gene Expression and Behavior of Smooth Muscle Cells in a 3D Culture Model. Front. Allergy.

[66] Devienne M.F., Guezennec C.Y. (2000). Energy expenditure of horse riding. Eur. J. Appl. Physiol..

[67] Weibel E.R., Taylor C.R., Hoppeler H., Karas R.H. (1987). Adaptive variation in the mammalian respiratory system in relation to energetic demand: I. Introduction to problem and strategy. Respir. Physiol..

[68] Ahern B.J., Sole A., de Klerk K., Hogg L.R., Vallance S.A., Bertin F.R., Franklin S.H. (2022). Evaluation of postsale endoscopy as a predictor of future racing performance in an Australian thoroughbred yearling population. Aust. Vet. J..

[69] Purcell S., Neale B., Todd-Brown K., Thomas L., Ferreira M.A.R., Bender D., Maller J., Sklar P., De Bakker P.I.W., Daly M.J. (2007). PLINK: A tool set for whole-genome association and population-based linkage analyses. Am. J. Hum. Genet..

[70] Biscarini F., Cozzi P., Gaspa G., Marras G. (2019). detectRUNS: Detect Runs of Homozygosity and Runs of Heterozygosity in Diploid Genomes. https://CRAN.R-project.org/package=detectRUNS.

[71] Lencz T., Lambert C., DeRosse P., Burdick K.E., Morgan T.V., Kane J.M., Kucherlapati R., Malhotra A.K. (2007). Runs of homozygosity reveal highly penetrant recessive loci in schizophrenia. Proc. Natl. Acad. Sci. USA.

[72] Purfield D.C., Berry D.P., McParland S., Bradley D.G. (2012). Runs of homozygosity and population history in cattle. BMC Genet..

[73] Goszczynski D., Molina A., Terán E., Morales-Durand H., Ross P., Cheng H., Giovambattista G., Demyda-Peyrás S. (2018). Runs of homozygosity in a selected cattle population with extremely inbred bulls: Descriptive and functional analyses revealed highly variable patterns. PLoS ONE.

[74] Huber W., Carey V.J., Gentleman R., Anders S., Carlson M., Carvalho B.S., Bravo H.C., Davis S., Gatto L., Girke T. (2015). Orchestrating high-throughput genomic analysis with Bioconductor. Nat. Methods.

[75] Lawrence M. (2023). HelloRanges: Introduce *Ranges to Bedtools Users. R Package Version 1.28.0. https://bioconductor.org/packages/HelloRanges.

